# Omitting transesophageal echocardiography before catheter ablation of atrial fibrillation

**DOI:** 10.1007/s10840-024-01825-8

**Published:** 2024-05-18

**Authors:** Vera Maslova, Thomas Demming, Robert Pantlik, Tamas Geczy, Peter Falk, Bjoern Andrew Remppis, Derk Frank, Evgeny Lian

**Affiliations:** 1 Department of Internal Medicine III, Cardiology and Angiology, University Hospital Schleswig-Holstein, Kiel, Germany; 2 Department of Cardiology, Cardiovascular Center Bad Bevensen, Bad Bevensen, Germany

**Keywords:** Ablation, Atrial fibrillation, Transesophageal echocardiography, Stroke

## Abstract

**Background:**

Data about necessity of performing transesophageal echocardiography (TOE) prior to every catheter ablation (CA) of atrial fibrillation (AF) is scarce. We aimed to evaluate the safety of an individualized risk-based approach to TOE with respect to thromboembolic cerebrovascular events (CVE) in patients undergoing CA for AF or left atrial tachycardia (AT).

**Methods:**

We performed a retrospective clinical study based on our institutional registry database. Patients undergoing CA for AF or left-sided AT following initial AF ablation at two participating centers were enrolled. Prior to the procedure, patients were scheduled for TOE only if they had a history of thromboembolic stroke, left atrial appendage (LAA) thrombus, or inappropriate anticoagulation regimen in the previous 3 to 4 weeks. The incidence of periprocedural cerebrovascular thromboembolic events was assessed.

**Results:**

We analyzed 1155 patients (median age 70 years, 54.8% male, 48.1% had persistent AF/AT). In 261 patients, a TOE was performed; in 2 patients (0.7%), an LAA thrombus was detected, which led to cancellation of the catheter ablation; in 894 patients, the TOE was omitted. Of the 1153 (0.35%) patients who underwent ablation, 4 (0.35%) experienced a CVE (one TIA and three strokes). The rate of CVE in our study does not exceed that reported in most multicenter trials. The low event rates limited statistical analysis of possible risk factors for CVE. In all 4 patients with CVE, post-CVE imaging showed the absence of LAA thrombus.

**Conclusions:**

An individualized selective approach to TOE before catheter ablation of AF or left AT showed a very low risk of overt intraprocedural thromboembolic events for the population in our study. A further randomized controlled study is needed to determine whether TOE prior to catheter ablation without ICE could be omitted in patients with uninterrupted OAC without previous thromboembolic events or a history of left atrial thrombus.

## Introduction

Atrial fibrillation (AF) is the most common cardiac arrhythmia in adults worldwide [1]. Catheter ablation (CA) is widely accepted as a safe and effective therapy for AF and left-sided atrial tachycardia (AT) and is an established standard of care.

However, the procedure can lead to life-threatening complications. One devastating but potentially preventable complication is a thromboembolic cerebrovascular event (CVE) [2]. Such events may result from mobilization of a left atrial appendage thrombus (LAAT) directly by catheter manipulation within the left atrium or because of adequate mechanical atrial contraction after restoration of sinus rhythm. Therefore, it is absolutely contraindicated to perform for CA of AF [2].

Transesophageal echocardiography (TOE) is the gold standard for pre-procedural screening for LAAT [2] and is commonly performed prior to the CA of AF [2]. However, concerns have been raised about the inherent risks of TOE, its cost-effectiveness, the prolonged procedural duration, and the increasing burden placed on echocardiography laboratories as the volume of procedures increases worldwide.

Uninterrupted periprocedural anticoagulation with either warfarin or novel oral anticoagulants (NOACs) is an effective therapy to minimize CVE risk [3–6]. Therefore, the need to perform TOE before every AF ablation remains controversial due to the limited available data.

Current guideline recommendations are also limited concerning the precise role of LA imaging for thrombus before CA, as no randomized clinical trials have been conducted on this subject [7]. Considering this, the recent European Heart Rhythm Association (EHRA) survey revealed significant variability in the use of TOE, with only 15% of centers conducting TOE before CA of AF in every patient [8]. In contrast, other institutions adopted a selective approach, considering the risk factors for LAAT in each patient.

Numerous studies have reported various predictors associated with an increased risk of LAAT, but data is still insufficient and does not propagate any widespread application in clinical practice [9–16].

Our study aimed to evaluate the safety of our individualized risk-based TOE approach for patients undergoing CA of AF or left-sided AT in the two participating centers.

## Methods

### Study cohort

We performed a retrospective clinical study based on our institutional registry database. Consecutive patients who were scheduled for CA as the treatment of AF or left-sided atrial tachycardia following initial AF ablation, from August 2018 to October 2022 in Heart Centre Bad Bevensen and at the University Hospital Schleswig-Holstein, Campus Kiel, were enrolled. The local ethic committee approved the study.

### Definitions

Periprocedural CVE was defined as the acute onset of a new neurological deficit between the start of the procedure and up to 24 h after ablation. Stroke was defined as a neurological deficit with an acute infarction on imaging. Transient ischemic attack (TIA) was defined as a neurological deficit that resolved within 24 h of onset by the absence of an acute infarction on imaging.

### Periprocedural anticoagulation

**Fig. 1:**
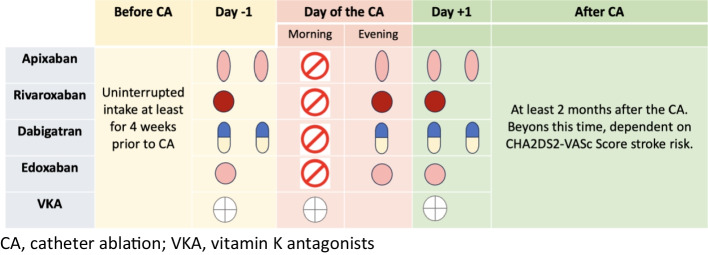
Periprocedural anticoagulation management

Most patients were anticoagulated with NOAC or vitamin K antagonist (VKA) for at least 4 weeks prior to ablation. In patients treated with NOAC, NOAC was discontinued in the morning before the procedure and resumed in the evening of the ablation day. Patients on apixaban or dabigatran received their usual dose in the evening. Patients on rivaroxaban and edoxaban received the full daily dose in the evening (Fig. 1). In patients receiving VKA, anticoagulation was not interrupted during the ablation procedure. Adherence to the anticoagulation regimen was assessed by verification of the international normalized ratio (INR) throughout the last 3 months for patients receiving VKA or by verbal communication for patients receiving NOAC. All patients with implanted LAA-occluders received therapy with ASS only.

All ablation procedures were performed under conscious sedation with propofol and fentanyl. For RF ablation, a double transseptal puncture was performed, one for the ablation catheter and the second for the diagnostic multielectrode catheter. For cryoballoon ablation, only a single transseptal puncture was performed.

Careful standard anticoagulation management was performed during the ablation procedure. Patients received an intravenous heparin bolus (100 IE per 1 kg of body weight) before the transseptal puncture with a target activated clotting time of 300 s. The transseptal introducer, ablation, and mapping catheters were continuously flushed with heparinized saline.

### Scheduling for transesophageal echocardiography

**Fig. 2:**
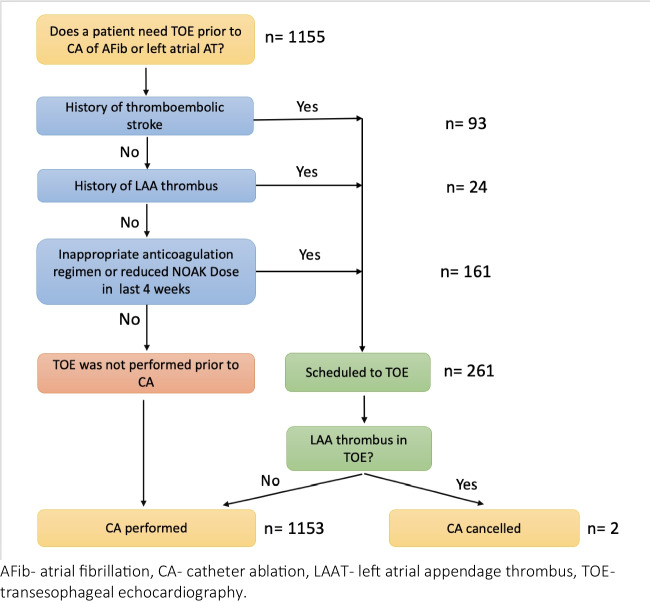
Risk-based scheduling to preprocedural TOE

Patients were eligible for TOE if one of the following criteria was met: (1) prior history of either thromboembolic stroke or (2) left atrial thrombus, (3) inappropriate anticoagulation regimen. The anticoagulation regimen was considered inappropriate if the patient was not anticoagulated (in patients without an LAA occluder or LAA resection) or if the NOAC was interrupted ≥ 1 missed dose in 3 weeks prior to CA or if the NOAC dose was reduced (including appropriately reduced dose). Anticoagulation with vitamin K antagonists (VKA) was considered inappropriate if the INR was < 2 within 3 months before ablation. Otherwise, no TOE was performed prior to CA (Fig. 2). All TOEs were performed on the day of the procedure immediately before the ablation.

### Post-procedural follow-up

Neurological status was checked immediately after the patient woke up. After CA, patients were monitored overnight in the hospital until the following day and discharged after clinical evaluation. Patients with CVE underwent imaging with computed tomography (CT) or magnetic resonance imaging (MRI) and were transferred to a stroke unit.

### Statistical analysis

Continuous variables are reported as the median and interquartile range (25–75%) where appropriate. Categorical variables were reported as percentages. The univariate comparison was performed using the *χ*^2^ or Fisher exact test for categorical variables, the *t*-test for continuous variables with uniform distribution, and the Mann-Whitney *U* test for those with non-uniform. A *p* value less than 0.05 (2-tailed) was considered statistically significant. Analyses were performed using Python 3 programming language, including the Pandas, Scipy, and Seaborn packages.

## Results

### Characteristics of the population

The baseline characteristics of the 1155 patients scheduled for ablation procedure for the treatment of AF or post-ablation left-sided AT and included in the study are summarized in Table 1. The median age was 70 (IQR, 62–76) years, and 674 patients (58.4%) were male. Overall, 555 (48.1%) patients had a persistent AF/AT and 357 (31%) had already previous left atrial CA, including 141 (12.2%) at one of the study centers. The CHA2DS2-VASc score was ≥ 2 in 938 (81.2%) patients. Preprocedural anticoagulation was used in 1108 (95.9%) patients with either NOAC therapy with apixaban (*n* = 530; 45.8%), rivaroxaban (*n* = 359; 31.1%), dabigatran (*n* = 22; 1.9 %), edoxaban (*n* = 103; 8.92%), or VKA therapy (*n* = 94; 8.14%). There were 161 (13.9%) patients without OAK or with inappropriate OAK intake/reduced dose before ablation (including six patients due to previous LAA occluder implantation and one patient due to previous LAA resection). Among patients with LAA occluders, all six patients received the device due to a history of life-threatening gastrointestinal bleeding. The median CHA2DS2-VASc score of these patients was 3.5 (IQR, 3–4.75). All patients with LAA occluder received antiplatelet therapy with acetylsalicylic acid. The median left ventricular ejection fraction was 59 (IQR, 50–60) %, and the median left atrial volume index was 43.7 (IQR, 32.7–55) ml/m^2^.  

**Table 1:** Baseline characteristics of patients

Characteristic	Total (*n* = 1155)		No TOE (*n* = 894)	TOE (*n* = 261)	*p*-value
Demographics					
Age, years	70 (IQR, 62–76)		70 (IQR, 62–76)	69 (IQR, 60–77)	0.31
Male	674 (58.4%)		518 (57.9%)	156 (59.8%)	0.59
Medical history					
Persistent AF/AT	555 (48.1%)	420(46.9%)		135 (51.7%)	0.17
Duration of AF, years	3 (IQR, 1–5)	3 (IQR1–5)		2 (IQR, 1–5)	0.24
AF on presentation	647 (56%)	497 (55.6%)		150 (57.4%)	0.59
Prior ablation in LA	357 (31%)	301 (33.7%)		57 (21.8%)	0.00027
BMI, kg/m^2^	28.07 (IQR, 25–31.7)	28 (IQR, 25–31)		28 (IQR, 25–31)	0.25
Hypertension	988 (85.6%)	773 (86.5%)		215 (82.3%)	0.09
Diabetes mellitus	170 (14.7%)	134 (15%)		36 (13.8%)	0.63
Coronary artery disease	275 (23.8%)	227 (25.4%)		48 (18.4%)	0.019
Obstructive sleep apnea	148 (12.8%)	117 (13.1%)		31 (11.9%)	
Impaired kidney function (GFR < 60)	207 (17.9%)	146 (16.3%)		61 (23.2%)	0.009
Previous CVE	93 (8.1%)	0		93 (35.6%)	< 0.00001
History of LAAT	24 (2.07%)	0		24 (9.2%)	< 0.00001
CHA2DS2-VASc score = 0	34 (2.94%)	21 (2.3%)		13 (5%)	0.02
CHA2DS2-VASc score = 1	183 (15.84%)	136 (15.2%)		47 (18%)	0.27
CHA2DS2-VASc score ≥ 2	938 (81.2%)	737 (82.4%)		201 (77%)	0.48
CHA2DS2-VASc score	3 (IQR, 2–4)	3 (IQR, 2–4)		3 (IQR, 2–4)	0.81
Preprocedural echocardiographic parameters					
Left ventricular EF, %	59 (IQR, 50–60)	60 (50–70)		58 (48–70)	0.09
Left ventricular EF < 35%	175 (15.2%)	141 (15.7%)		34 (13%)	0.27
Left atrium volume index (ml/m^2^)	43.7 (IQR, 32.7–55)	43 (IQR, 32–54)		43 (IQR, 33–55)	0.92
Anticoagulation					
NOACs	1014 (87.8%	827 (92.5%)		177 (67.8%)	< 0.00001
Apixaban	530 (45.8%)	443 (49.659)		87 (33.3%)	< 0.00001
Rivaroxaban	359 (31.1%)	294 (32.95)		65 (22.3%)	0.014
Dabigatran	22 (1.9%)	14 (1.6%)		8 (2.7%)	0.119
Edoxaban	103 (8.92%)	86 (9.6%)		17 (5.8%)	0.12
VKAs	94 (8.13%)	62 (6.9%)		32 (12.3%)	0.005
No or inappropriate intake/reduced dose of OAC 3 weeks before the procedure	161 (13.9%)	1 (0.1%)		160 (61.3%)	< 0.00001
LAA occluder	6 (0.52%)	4 (0.4%)		2 (0.6%)	0.52
LAA resection	1 (0.09%)	1 (0.1%)		0	1

*AF* atrial fibrillation, *AT *atrial tachycardia, *BMI* body mass index, *CVE* cerebrovascular event, *GFR* glomerular filtration rate, *EF* ejection fraction, *HFrEF* heart failure with reduced ejection fraction, *LA* left atrium, *LAA* left atrial appendage, *LAAT* left atrial appendage thrombus, *NOACS* non-vitamin K antagonist oral anticoagulants, *TOE * transesophageal echocardiography, *VKA* vitamin K antagonists

#### Comparison between patients with and without TOE

Preprocedural TOE was performed in only 261 (22.6%) patients. Ninety-three patients had a history of stroke, 24 had a history of left atrial appendage thrombus, and 160 were inappropriately anticoagulated. Most patients (77.4%) did not have TOE.

The characteristics of patients with and without TOE performed are summarized in Table 1, showing the difference in some parameters. There were significantly more patients with renal insufficiency in the TOE group, which was expected due to the TOE scheduling of all patients with reduced NOAC dose (the main reason for the reduction was impaired renal function). Also, significantly more patients on VKA received TOE, indicating that these patients had difficulties maintaining their INR. Patients in the TOE group had a lower prevalence of coronary artery disease and prior ablation in the left atrium (more patients with prior ablation in LA in the group without TOE). Other parameters were not statistically different.

LAA thrombus was detected in 2 out of 261 (0.76%) patients scheduled for the TOE, and the ablation procedure was cancelled (Table 2).

**Table 2:** The clinical characteristics of the patients with LAA thrombus detected

	Patient A	Patient B
Age, years	75	69
Gender	Female	Male
BMI, kg/m^2^	28	28.9
CHA2DS2-VASc score	3	3
OAK substance and regimen	Apixaban 5 mg twice daily	Rivaroxaban 15 mg
LVEF, %	55	25
LAVI, ml/m^2^	48.2	76.4
GFR	90	52
AF type	Persistent	Persistent
Presenting rhythm	AF	AF
History of CVE	No	No
Reason for TOE	Inappropriate NOAC regimen	Inappropriate NOAC regimen
History of LAAT	No	No
LAA occluder	No	No

Abbreviations as in Table 1

### Cerebrovascular events

In total, 4 out of 1153 (0.35%) patients who underwent ablation procedure experienced thromboembolic CVE (one TIA and three strokes). The clinical characteristics of these patients, as well as their anticoagulation regimen and event characteristics, are summarized in Table 3. Two strokes occurred in patients who underwent cryoablation; one stroke and one TIA occurred after RF ablation. None of the patients who experienced a stroke had an LAA occluder. One of the patients with stroke underwent mechanic thrombectomy of the arteria cerebri media. The other three patients received only conservative treatment. Fortunately, all patients were free of neurological deficits at hospital discharge.

**Table 3:** The clinical and procedural characteristics of the patients with CVE

	Patient #1	Patient #2	Patient #3	Patient #4
Age, years	69	80	71	66
Gender	Female	Female	Male	Female
BMI, kg/m^2^	33.66	33.28	25.01	23.95
CHA2DS2-VASc score	3	4	5	3
CHADS2 score	1	2	4	1
LAA occluder	No	No	No	No
OAK substance and regimen	Apixaban, 5 mg, twice daily	Apixaban, 2.5 mg, twice daily	Apixaban, 5 mg, twice daily	Rivaroxaban, 20 mg, once daily
LVEF, %	37	60	44	60
LAVI, ml/m^2^	53.8	66.8	-	-
AF type	Paroxysmal	Paroxysmal	Paroxysmal	Persistent
Presenting rhythm	SR	SR	Afib	AFib
TOE performed	No	No	Yes	Yes
History of CVE	No	No	No	No
Reason for TOE	-	-	Inappropriate OAK intake	Inappropriate OAK intake
History of LAAT	No	No	No	No
No or inappropriate intake/reduced dose of OAC 3 weeks before the procedure	Yes	No	Yes	Yes
Procedure type	RF PVI	Cryo-PVI	RF PVI	Cryo-PVI
Type of CVE	Thromboembolic	Thromboembolic	Thromboembolic	Thromboembolic
Time of CVE diagnosis	Periprocedural	Periprocedural	Periprocedural	Periprocedural
Post-procedure imaging	No LAA thrombus	Sludge in LAA	No LAA thrombus	No LAA thrombus

Abbreviations as in Table 1

In two cases of periprocedural CVE, TOE was performed before CA (due to inappropriate NOAC intake: more than one missed dose in 4 weeks before CA), and LAA thrombi were ruled out. We suggest that the etiology of stroke in patients without pre-existing LAA thrombus is the thrombus formation on the catheters or sheaths, as no apparent source of the air embolism was detected during the procedure. In  further two cases, TOE was not performed. Two patients had no indication for TOE according to our individualized approach, although one of them admitted an inappropriate NOAC regimen, but only after CA had been performed.

A comparison of the two groups (with and without CVE) is also summarized in Table 4. 

**Table 4:** Univariate comparisons of patient characteristics between patients undergone CA with and without CVE in the periprocedural period

	No CVE (*n* = 1149)	CVE (*n* = 4)	*p* value
Age, years	70 (IQR, 62–76)	70 (IQR, 68–73)	0.58
Male sex	673 (58.5%)	1 (25%)	0.31
BMI, kg/m^2^	28.06 (IQR, 25.01–31.70)	29.1 (IQR, 24.7–33.4)	0.85
Persistent AF	554 (48.2%)	1 (25%)	0.63
Prior ablation	355 (30.8%)	0	0.32
AF on presentation	645 (56.1%)	2 (50%)	1
Duration of AF, years	3 (1–5)	1.5 (1.25–1.75)	0.31
CHA2DS2-VASc score	3 (2–4)	3 (2.5–3.5)	0.1
CHA2DS2-VASc score ≥ 2	935 (81.3%%)	3 (75%)	0.57
Anticoagulation with DOAC	985 (85.7%)	4 (100%)	1
No or inappropriate intake/reduced dose of OAC 3 weeks before the procedure	232 (20.2%)	2 (50%)	0.18
History of LAAT	24 (2.1%)	0	1
Previous CVE	93 (8.1%)	0	1
Coronary artery disease	274 (23.8%)	1 (25%)	1
Diabetes mellitus	168 (14.6%)	2 (50%)	0.11
LAVI (ml/m^2^)	43 (32–54)	Mean 60.3	0.45
LVEF < 45%	269 (23.4%)	2 (50%)	0.24
LVEF, %	59 (IQR, 50–60)	52 (IQR, 42–61)	0.79

Abbreviations as in Table 1

The clinical characteristics of patients suffering periprocedural stroke were similar to those of patients without CVE. Statistical comparison with the patients without CVE was limited by sample size.

In all four patients with CVE, the imaging after CVE (TOE was performed in the first 48 h) showed the absence of LAAT.

Even though not statistically significant, the higher rate of the CVE in the patients who underwent TOE (two out of 261 patients, 0.76%) highlights the higher baseline risk of thromboembolic events in this group of patients compared to the low-risk patients who did not undergo TOE (two out of 895 patients, 0.22%, *p* = 0.22).

## Discussion

We evaluated safety of our individualized approach to pre-procedural TOE in patients undergoing CA of AF or left-sided ATs at two centers over 4 years. Our approach was to perform TOE only in patients with inappropriate anticoagulation regimens or a history of prior thromboembolic CVE or LAAT. To our knowledge, this is the first large prospective study to describe a selective approach regarding preprocedural TOE.

The major findings were as follows: (1) Our individualized risk-based approach for TOE planning proved to be safe with a stroke incidence of 0.35%, which was not higher than in large cohort studies [6, 17, 18]; (2) stroke cases in patients without pre-existing LAAT indicate possible intraprocedural thrombus formation on catheters or transseptal sheaths. Due to the low incidence of stroke (four out of 1155 patients), no statistically significant predictors for periprocedural stroke could be identified.

### Mechanism of periprocedural thromboembolism

There are four possible scenarios of thromboembolism during and immediately after catheter ablation in the left atrium:

1. Mobilization of pre-existing LAA thrombus by catheter manipulations
2. Cloth formation on the catheter and transseptal sheaths
3. Cloth formation on the endothelium injured by ablation
4. Mobilization of a thrombus formed at the femoral vein access site with passage through the created atrial septal defect

The main mechanism of massive thromboembolic CVE during and after the CA of AF is the dislodgement of a preformed thrombus, mainly from the LAA [19]. To prevent CVE in this scenario, we want to ensure that there is no thrombus in the LAA prior to intervention. This can be achieved by (1) thorough anticoagulation for a minimum of 3 weeks, which prevents thrombus formation and dissolves existing thrombi, or by (2) performing TOE.

However, it remains unclear whether it is necessary to perform TOE before all left atrial percutaneous procedures to minimize periprocedural risk of stroke or whether TOE could be omitted in selected cases without increasing the incidence of stroke. Furthermore, we should not forget that stroke can occur in patients without pre-existing LAA thrombus. The study by Page et al. reported about 0.4% of strokes (five strokes in 1202 patients) in patients who underwent CA of AF despite anticoagulation with continuous warfarin with therapeutic INR and LAAT exclusion in TOE [20]. Therefore, intraprocedural anticoagulation and transseptal sheath management are as important as the exclusion of preformed thrombus in LAA [21–23].

#### What speaks against performing TOE in every patient?

TOE is generally considered to be safe and relatively non-invasive. However, it can cause serious and potentially life-threatening complications, such as perforation, severe bleeding, and aspiration. That is why the risks of TOE need to be balanced against its benefits as a diagnostic tool [24]. In a large review representing 42,355 patients, Cote et al. reported a perforation rate of 0.02 to 0.2% for diagnostic procedures [25]. The incidence of bleeding is reported to be 0.02 to 0.03% [26, 27]. Incorrect insertion of the esophageal probe into the trachea has been reported in 4 out of 1500 examinations (0.27%) in ambulatory adults, which can lead to severe pneumonia [28]. Although the likelihood of TOE leading to a major complication is low, the possibility does exist. There are also major concerns about cost-effectiveness, prolonged procedure times, and the growing burden placed on echocardiography laboratories as the volume of procedures increases worldwide [29]. These facts argue for a selective approach to TOE based on the likelihood of finding an LAA thrombus. In our study cohort, no TOE-related complications were observed in 261 patients.

#### What is the likelihood of finding an LAA thrombus on TOE?

It depends on the anticoagulation regimen (Table 5).

**Table 5:** A comparison of studies reported the prevalence of LAA thrombus on different anticoagulation regimens

	Number of patients				LAA-thrombus detected				Adherence	
	Overall	No OAC	On VKA	On NOAC	Overall	No OAC	On VKA	On NOAC	To NOAC	To VKA
The ACUTE trial, 2002 [30]	1152	549 (47.6%)	603 (52.3%)	-	76 (13.8%) TOE performed only in no OAC group!	76 (13.8%)	No data	-	-	Not reported
Puwanant et al. 2009 [31]	1058	283 (26.7%)	775 (73.3%)	-	6 (0.6%)	0	6 (0.8%)	-	-	Not reported
Milhem et al. 2019 [9]	2494	235 (9.4%)	1059 (42.5%)	1186 (47.6%)	48 (1.92%)	2 (0.8%)	27 (2.5%)	19 (1.6%)	Not reported	18 of 27 patients with LAA thrombus
Alqarawi et al. 2019 [32]	942	0	496 (53%)	447 (47%)	3 (0.3%)	0	3 (0.6%)	0	Not reported	Not reported
Wegner et al. 2022 [33]	512	82 (16%)	138 (27%)	292 (57%)	19 (3.7%)	3 (3.6%)	7 (5.1%)	9 (3.15)	< 3 weeks before or uncertain adherence	< 3 weeks before or uncertain adherence

*CA* catheter ablation, *VKA* vitamin K antagonists, *AFib* atrial fibrillation, *CA* catheter ablation, *LAAT* left atrial appendage thrombus, *TOE* transesophageal echocardiography

Several studies reported the highest prevalence of LAA thrombus detection in patients without OAC (up to 13.8%), followed by patients on VKA (0.3–2.5%) and the lowest incidence in patients on NOAC (0–3.2%) [30]. Unfortunately, not all these studies reported patients’ adherence to OACs. Nevertheless, we can observe that the likelihood of LAA thrombus formation decreases over time with improved OAC management. This is especially the case after the introduction of NOACs when more patients permanently remain in the therapeutic anticoagulation range compared to VKA. Moreover, repeated TOE is unlikely to detect LAA thrombus in patients without LAA thrombus in the baseline study [34]. All this prompted us to adopt a selective approach to TOE in patients already receiving anticoagulation. Our study’s detection rate of LAA thrombus was 0.76% in the cohort of high-risk patients who met the criteria for scheduling for TOE.

### Selection criteria for TOE

TOE was performed in all patients who were on OAC or had an inappropriate regimen (discontinued or low dose) 3 weeks prior to the ablation procedure. In addition, we considered patients at high risk of LAA thrombus formation if they were on OAC and had:

1. History of LAA thrombus
2. History stroke or another thromboembolic event
3. Reduced NOAC dose
4. In case of VKA: INR was < 2 within 3 months before ablation

The reasons for including these criteria and avoiding others are the following. As mentioned above, patients with *a history of LAA thrombus* are more likely to have one detected at repeated TOE sessions [34]. As the use of OAC reduces the risk of CVE in patients with high CHA2DS2-VASc, we used only the *history of stroke* as the risk factor with the highest impact (2 points), but not the whole score for planning TOE [35]. We performed TOE in all patients on *reduced NOAC dose* for two reasons. First, most NOAC safety studies were performed with full-dose NOAC [36]. Second, to exclude the risk of patients receiving inappropriately reduced dose of NOAC [37].

Criteria such as rhythm at the time of the procedure, paroxysmal vs. persistent AF, and AF burden were avoided in the decision-making process as more and more data support the temporal dissociation between the CVE and the AF episodes [38]. These data support the suggestion that AF as a cause of the stasis in Virchow’s triad may not have such a significant impact on thrombus formation but is only an indicator of underlying structural conditions (atriopathy, fibrosis, endothelial dysfunction) that play a much more important role in thrombogenesis. Studies conducted in the era of VKA and preprocedural bridging with fractionated heparin have identified several risk factors for LAA thrombus or ischemic stroke in patients on warfarin in addition to those already included in CHA2DS2−VASc [39]. Conditions such as increased LA size, cardiomyopathy with low LVEF, D-dimer levels, hyperuricemia, and hypertrophic cardiomyopathy have only been associated with CVE in non-randomized studies in the VKA era [10, 11].

#### Safety of catheter ablation with selective TOE screening

This is the first study to report safety data on selective TOE screening before catheter ablation of AF or left-sided AT. Omission of TOE prior to CA of AF has been demonstrated in patients on continuous NOACs using intracardiac echocardiography, which can be used as a tool to visualize the LAA [29, 40]. Diab et al. reported the safety of catheter ablation of atrial fibrillation in NOAC-compliant patients without TOE screening with a low rate of thromboembolic events (0.3%) [41]. But again, the authors used ICE imaging throughout the procedure in all patients, even though they claimed not to use it explicitly for LAA visualization from the right ventricular outflow tract.

Despite the non-randomized design of our study, it demonstrates the safety of CA for AF with selective TOE screening. The rate of CVE in our study (0.35%) does not exceed that reported in multicenter studies (0.3–0.5%) where all patients underwent TOE screening [42, 43]. Selective preprocedural TOE detected LAAT in 2 out of 261 patients (0.76%). In 2 out of 4 patients with periprocedural CVE, the LAAT was excluded by TOE screening indicating possible intraprocedural thrombus formation on the catheters or transseptal sheaths. Interestingly, in all patients with stroke, post-stroke LAAT was excluded on imaging. However, one had sludge in LAA, and the other might have had mobilized the thrombus during catheter manipulation. Our data show that preprocedural TOE cannot prevent all thromboembolic events because a significant proportion is unrelated to the pre-existing thrombus.

The authors of a meta-analysis including 16 clinical trials found no difference in CVE between cryoballoon technology and radiofrequency ablation [44]. In our study, 2 patients with CVE received radiofrequency ablation and another 2 received cryoballoon ablation. Due to the low incidence of CVE in our study, we could not identify any factors statistically associated with periprocedural CVE (Table 4).

#### The implication for current practice in performing TOE

Guidelines are limited on the precise role of LA imaging for thrombus before CA. There are no randomized trials on this topic. The 2019 AHA/ACC/HRA focused update of the 2014 AHA/ACC/HRS Guidelines for the management of patients with atrial fibrillation recommends that TOE should be considered in patients even if they are receiving OAC for more than 3 weeks before ablation [45]. The current ACC Guidelines 2023 recommend performing CA of AF on uninterrupted NOAKs or VKA; the role of the preprocedural TOE is not mentioned [46]. The 2020 ESC guidelines for diagnosis and management of atrial fibrillation give a class IIa (level of evidence C) recommendation for either therapeutic anticoagulation within 3 weeks before ablation or TOE to exclude LA thrombi [7].

Balouch et al., assessing the trends in the use of TOE, reported a decrease in preprocedural use of TOE from 86% in 2010 to 42% in 2015, with no change in the incidence of CVE during the study period [11]. The 2019 EHRA survey revealed great variability in the use of TOE before left-sided CA [8]. Only 15% of centers perform TOE before CA of AF in all patients, whereas other centers use a selective approach, depending on the risk factors of each patient. Most commonly, TOE was performed in cases of inadequate or unclear preprocedural anticoagulation cases, even in AF lasting < 48 h.

For the first time, our data demonstrates the safety of performing CA of AF with selective TOE screening even without the use of ICE. The current study confirms that the number of patients undergoing preprocedural TOE can be significantly reduced without an increase in periprocedural adverse events. An individualized approach to TOE may significantly reduce the number of complications associated with TOE and the economic burden on the healthcare system. By reporting the results of this approach, we hope to provide valuable insights into refining preprocedural screening practices and optimizing patient safety during CA procedures.

### Limitations

The main limitation of the study is its non-randomized nature, where we analyzed a population with particular characteristics in a retrospective manner, not systematically assessing for the presence or absence of thrombus or cerebral embolism. Hence, the conclusion is that an individualized selective approach to omit TOE in patients without previous thromboembolic events or left atrial thrombus is only valid in this specific analyzed population and only for the endpoint of overt stroke. Furthermore, the low incidence of stroke does not allow us to identify any predictors of stroke. Because we did not perform intracardiac or transesophageal ultrasound monitoring during catheter ablation, we cannot rule out clot formation on the catheters or the transseptal sheath as a source of embolism.

Also, our study does not include data on silent cerebral embolic events (SCEEs), which could emerge after the CA of AF even in patients under continued oral therapeutic anticoagulation [47]. SCEEs are important because of their association with dementia and cognitive decline in the general population [48, 49]. The silent cerebral lesions can be detected only with pre-procedural and post-procedural cerebral MRI, which was not routinely performed in our patient group. That is why we cannot exclude with the current data that other parameters may play a central role in the need for TOE before CA. A further randomized study is needed to ensure that the observation in our study would validate in a population with other structures of baseline clinical characteristics, such as a higher CHADSVASC score, age, and comorbidities.

## Conclusions

An individualized selective approach to TOE before catheter ablation of AF or left AT showed a very low risk of overt intraprocedural thromboembolic events for the population in our study. A further randomized controlled study is needed to determine whether TOE prior to catheter ablation without ICE could be omitted in patients with uninterrupted OAC without previous thromboembolic events or a history of left atrial thrombus.

## Data Availability

Not applicable.
